# Prenatal diagnosis of 4953 pregnant women with indications for genetic amniocentesis in Northeast China

**DOI:** 10.1186/s13039-019-0457-x

**Published:** 2019-11-06

**Authors:** Rulin Dai, Yang Yu, Qi Xi, Xiaonan Hu, Haibo Zhu, Ruizhi Liu, Ruixue Wang

**Affiliations:** Department of Reproductive Medicine, Department of Prenatal Diagnosis, First Hospital, Jilin University, 71 Xinmin Street, Changchun, 130021 Jilin Province People’s Republic of China

**Keywords:** Prenatal diagnosis, Advanced maternal age (AMA), Non-invasive prenatal testing (NIPT), Ultrasonography, Fluorescence in situ hybridization (FISH)

## Abstract

**Background:**

Several different technologies are used for prenatal screening procedures and genetic diagnostic technologies. We aimed to investigate the rates of chromosomal abnormalities in cases with different abnormal prenatal indications and to determine the relationships between fetal chromosomal abnormalities and indicators of prenatal abnormalities in Northeast China.

**Methods:**

We evaluated 4953 16- to 23-week singleton gestation cases using amniocentesis and a total of 3583 participants received serological screening. Fetal chromosomal analyses were performed for all samples using fluorescence in situ hybridization and karyotyping.

**Results:**

Among these samples, 204 (4.12%) had fetal chromosomal abnormalities. A total of 3583 participants received serological screening, among whom 102 (2.85%) exhibited positive results. A total of 309 participants had ultrasonography; 42 (13.6%) of these had abnormalities. Among 97 participants who had non-invasive prenatal testing (NIPT), 59 (61%) had positive results. Among 1265 participants with advanced maternal age, 78 (6.2%) had abnormal results.

**Conclusion:**

The serological screening and NIPT that were included in the prenatal screening methods all had false positive and false negative rates. Although they are both prenatal screening techniques, maternal serum screening cannot be replaced by NIPT. The pregnancy women should accept NIPT in a qualified prenatal diagnostic center. We recommend that pregnant women at high or critical risk undergoing prenatal screening should confirm the fetal karyotype through amniocentesis. Moreover, if women receive a positive result via NIPT, they should not have a pregnancy termination without undergoing further prenatal diagnosis.

## Introduction

Several different technologies are used for prenatal genetic screening procedures and diagnostic technology, including ultrasonography, the double-marker test, the triple marker test, non-invasive prenatal testing (NIPT) [1]. Invasive prenatal diagnostic techniques are feasible tools for confirming fetal chromosomal abnormalities [2]. Amniocentesis is a reliable and low-risk method of achieving suitable genetic material [3]. Application of this technique to amniotic fluid analysis can enable physicians to recognize fetal genetic abnormalities. Routine chromosome analysis has been the gold standard for prenatal cytogenetic diagnosis [4]. However, karyotyping has a limitation in that embryonic cell culture takes about 2 weeks or even longer. These long waiting times for karyotyping results can cause much psychological distress to the couples.

At present, fluorescence in situ hybridization (FISH) has been used for rapid prenatal diagnosis of the most common aneuploidies in pregnant woman at high risk. Down’s syndrome (DS, trisomy 21) and Edwards syndrome (ES, trisomy 18) are two most prevalent autosomal trisomies encountered at birth. The main advantages of FISH technology are that it is rapid and reliable [5, 6].

Non-invasive analysis of fetal genetic traits using material blood samples has been commonly used for the prenatal diagnosis of DS [7]. Lo et al. [8] first reported the presence of fetal DNA in maternal blood. Then, NIPT was initially conducted by DNA sequencing techniques [9, 10]. Many physicians dealing with high-risk pregnant women consider this to be one of the safest methods in the detection of fetal aneuploidies [11].

Nuchal translucency (NT) testing in combination with measurements of pregnancy-associated plasma protein A and the free β subunit of human chorionic gonadotropin (hCG) have been included in prenatal screening options during the first trimester. In the second trimester, serum monitoring using triple screening (free β-hCG, maternal serum alpha fetoprotein (AFP), and unconjugated estradiol levels) or quadruple screening (hCG, inhibin A, maternal serum AFP, and unconjugated estradiol levels), and ultrasonography have been included [12, 13].

Ultrasonography is generally considered to be a safe method of imaging, often used for diagnosing fetal congenital disabilities or abnormal development. Thus, observing fetal NT values and nose bone development could allow a prediction of the possibility of the fetus having DS. However, this technology has a high false positive rate of 5% [14]. In addition to detecting DS, an increased NT have been described in association with other genetic fetal syndromes like 22q11 microdeletion syndrome, Noonan syndrome, Smith-Lemli-Opitz syndrome, multiple pterygium syndrome (Escobar syndrome), Fanconi pancytopenia syndrome etc. [15–21].

Here we aimed to investigate the rates of chromosomal abnormalities in various abnormal prenatal conditions, and to determine the relationship between these and indicators of prenatal abnormalities in Northeast China.

## Results

All 4953 amniocentesis samples except for one (karyotyping showed trisomy 18; the FISH result was normal) were the same by FISH and karyotyping. Among the samples, 204/4952 (4.12%) had fetal chromosomal abnormalities. A total of 3583 participants received serological screening, among which 102 (2.85%) exhibited positive results. A total of 309 participants underwent ultrasonography and 42 (13.6%) had abnormalities. Among 97 participants who had NIPT, 59 (61%) had positive results. Of 1265 participants classed as women of AMA, 78 (6.17%) had abnormal results.

Many indicators in our study could reflect abnormal fetal development, such as positive serological and NIPT results, women of AMA, abnormal ultrasound findings and a history of abnormal pregnancies. The most frequent related indicator was a positive serological finding (102/204; 50%), followed by women of AMA (78/204; 38.2%), a positive NIPT (59/204; 28.9%) and abnormal ultrasound findings (42/204; 20.6%; Table 1).

**Table 1:** Maternal age and fetal chromosomal abnormality indicators

Abnormal indicators	≧40	35–39	30–34	25–29	< 25	Total, n
Positive serological	7	9	22	32	11	81
Positive NIPT	9	10	11	3	1	34
Abnormal ultrasound	3	7	11	15	0	36
Age factor	13	11	2	0	0	26
History of abnormal pregnancy	0	0	0	1	0	1
Positive NIPT+ Positive serological	3	4	2	7	4	20
Positive NIPT+ Abnormal ultrasound	1	0	0	3	1	5
Abnormal ultrasound+ Positive serological	0	1	0	0	0	1
Cases, n	36	42	48	61	17	204

**Fig. 1:**
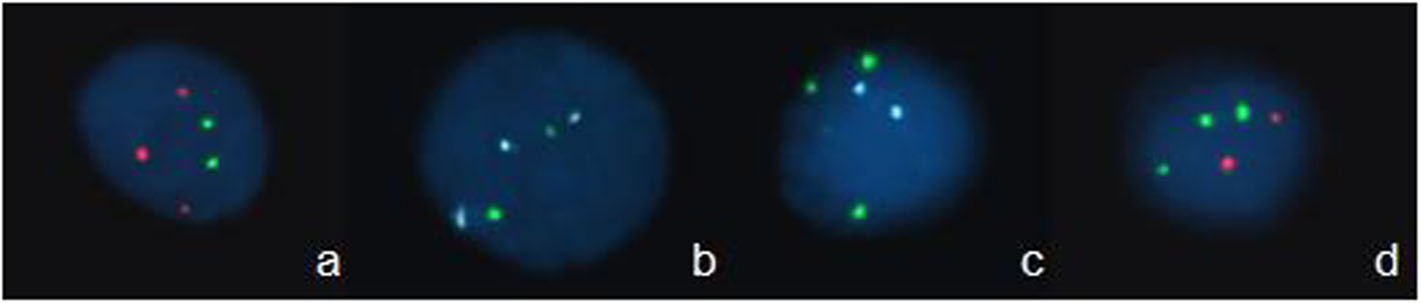
FISH results of the most common fetal chromosomal abnormalities. **a** Trisomy 21, **b** Trisomy 18, **c** XXX, **d** Trisomy 13

Among 204 abnormal samples, the most frequent karyotype was trisomy 21 (143/204; 70.1%), followed by trisomy 18 (33/204; 16.2%; Table 2, Fig. 1). Of the 204 abnormal fetal karyotypes, 97 (47.6%) had a positive NIPT. The true positive fetal karyotype by NIPT was 61% (59/97), however, the false positive rate was 39% (38/97). Trisomy 21 had the highest true positive rate by NIPT (47%, 46/97), however, this also had a higher false positive rate (19%, 18/97, Table 3).

**Table 2:** Maternal age and karyotype of fetal chromosomal abnormality

Fetal chromosomal karyotype	Cases, n	≧40	35–39	30–34	25–29	< 25
Trisomy 21	143	26	31	37	36	13
Trisomy 18	33	9	5	3	14	2
XXX	10	0	2	3	5	0
Trisomy 13	6	1	2	0	2	1
Monosomy X	5	0	0	3	2	0
Sex chromosome mosaicism	3	0	0	1	1	1
XYY	2	0	2	0	0	0
XXY	2	0	0	1	1	0
Total	204	36	42	48	61	17

**Table 3:** Fetal positive karyotype analysis of NIPT

Fetal karyotype	False positive, n(%)	True positive, n(%)
Trisomy 21	18(18.56%)	46(47.42%)
Trisomy 18	6(6.19%)	8(8.25%)
Trisomy 13	2(2.06%)	1(1.03%)
Sex chromosome abnormal	8(8.25%)	4(4.12%)
Other chromosome abnormal	4(4.12%)	0(0.00)
Total	38(39.18%)	59(60.82%)

Of the 102 positive serological samples, there were 20 cases with a positive NIPT result. Among these samples, 82 high-risk samples (incidence of DS ≥ 1:270 and of ES ≥ 1:350) had abnormalities, 20 reaching the critical point for high-risk samples (incidence of DS 1:270–1:1000 and of ES 1:350–1:1000) had abnormalities (Table 4).

**Table 4:** Positive serological and positive NIPT

	Cases, n	DS≧1:270 & ES≧1:350, n	DS 1:270~1:500 & ES 1:350~1:500, n	DS and ES 1:500~1:1000, n
Positive serological	102	82	16	4
Positive NIPT	20	14	4	2

Ultrasonography is used in determining many types of fetal abnormalities. These include increased NT, heart abnormalities, choroid plexus cysts, lymphatic hydroceles of the neck, skeletal and brain abnormalities, increased neck fold (NF), and kidney, bowel, and lung abnormalities. Ultrasonic findings of increased NT or NF values, and of skeletal or bowel abnormalities might indicate DS. followed by heart abnormalities (Table 5).

**Table 5:** Ultrasonic findings of abnormal fetal

Ultrasonography	Cases, n	Trisomy 21	Trisomy 18	Trisomy 13	Monosomy X	XXX
Increased NT	18	12	2	0	2	2
Heart abnormality	14	4	5	2	2	1
Choroid plexus cyst	6	0	6	0	0	0
Neck lymphatic hydrocele	6	1	1	0	3	1
Bone abnormality	6	4	1	1	0	0
Brain abnormality	5	1	2	2	0	0
Increased NF	4	3	0	0	0	1
Kidney abnormality	4	1	1	2	0	0
Bowel abnormality	2	2	0	0	0	0
Lung abnormality	2	1	0	0	0	1

## Discussion

At present, several different technologies are used for prenatal screening and diagnosis. Here we aimed to determine the relationships between fetal chromosomal abnormalities and prenatal indicators of fetal abnormalities in Northeast China.

The amniocenteses were for indications including AMA, a positive NIPT result, a positive aneuploidy screening result, abnormal ultrasound findings, a history of abnormal pregnancy, and paternal/maternal carriers of known genetic disorders. Among the samples, 204 (4.12%) had fetal chromosomal abnormalities. Among these, the most frequent indicators were a positive serological finding of possible abnormalities, followed by AMA, a positive NIPT and abnormal ultrasound observations.

The serological screening is the method determining a risk of fetal chromosomal disease. Recent studies have suggested that serological screening is an important marker related to fetal chromosomal aneuploidy, a single abnormal serum marker with ultrasound examination can facilitate screening for fetal chromosome abnormalities in pregnant women [22], and could increased the detection rate of fetal chromosomal aneuploidy through serological screening [23]. High-risk and critical-risk pregnant women subjected to prenatal screening in the first and second trimesters of pregnancy should undergo prenatal diagnosis. In the present study, among 102 positive serological samples, 20 that reached the critical point for risk had abnormalities. Therefore, we recommend that critical point for risk pregnant women should confirm fetal karyotypes by amniocentesis, which is often overlooked by people. The elderly pregnant women, who were at critical-risk and refused amniocentesis, underwent NIPT of fetal DNA upon recommendation. Although both are prenatal screening techniques, serological screening cannot be replaced by NIPT.

In our study, the most frequent abnormal karyotype was of DS (70.1%), followed by ES (16.2%). Non-invasive analysis of fetal genetic traits using material blood samples has been used commonly for the prenatal diagnosis of DS. However, we found true positive rate of NIPT for diagnosing fetal karyotypes was 60.82%, false positive rate was 39.18%. Trisomy 21 (DS) had the highest true positive rate by NIPT (47.42%); however, it also had a higher false positive rate (18.56%), followed by the false positive rate of sex chromosome abnormalities (8.25%). This is inconsistent with results published previously. Zhang et al. [24] reported that the accuracy and specificity of screening for DS in 87 women classed as being of AMA was 100%. Due to NIPT is rather newly introduced, and experiences with discordant results are few. Although NIPT is not done by our laboratory, clinical informations were directly obtained from pregnant women using the questionnaire. Our FISH results confirm that the sensitivity of NIPT technology is not so high. In 1997, cell-free fetal DNA fragments were discovered in the plasma of pregnant women, and in 2011, NIPT was introduced into clinic and commerce [25]. In view of a high sensitivity and specificity of NIPT, it is considerd as an incomparable screening test for fetal aneuploidy. However, in 2016, the American College of Medical Genetics and Genomics has released an important new policy statement that NIPS instead of NIPT, where the “S” represents screening, it should be highlighted that NIPT is not a diagnostic technique [11]. One should remember that the NIPT is only a screening test which provides a risk for the genetic disorder, but not the diagnosis. Many companies in the market can now do NIPT testing, and we recommend that pregnancy women should accept it in a qualified prenatal diagnostic center.

Some studies have discussed the effect of maternal age on spontaneous abortion and have suggested that AMA is an important factor related to fetal chromosomal aneuploidy, with aneuploidy rates increasing with age [26, 27]. We aimed to determine the relationship between abnormal fetal karyotype and AMA using amniocentesis. Therefore, we divided all samples with abnormal fetal karyotypes into five age groups according to maternal age. The fetal abnormality rate in 1265 women classed as AMA was 6.17%. Among abnormal fetal findings, AMA women accounted for 38.2%. We consider that physicians dealing with such AMA women should pay more attention to spontaneous abortion, as performing amniocentesis is still the best choice in the second trimester. Chromosome abnormalities usually result in developmental arrest and spontaneous abortion. Our laboratory historically [28] used FISH technology for testing chorionic villi, and found that the kinds of fetal abnormalities, numbers of abortions, and chromosomal abnormality rates increased with increasing maternal age, and we hypothesize whether the larger probability of chromosomal abnormalities is due to increased mutation rate with maternal age, or due to a worse in-utero conditions. FISH probes for abortion samples could detect more chromosomal numerical abnormalities for it can detect 16/22 addtionally. Chromosomal abnormalities in aborted fetus can more accurately reflect the fetal chromosomal anomalies in AMA women.

Ultrasonography is generally considered a safe and indispensable method of imaging in pregnancy, although it cannot detect genetic defects. More than 50 genetic conditions have been described in association with an increased NT [29, 30]. Pregnant woman with a fetal NT of > 3.0 mm indicating DS are deemed as having an abnormal pregnancy and these were the most frequent in our study. Other abnormal ultrasonic results included heart abnormalities, choroid plexus cysts, lymphatic hydroceles in the neck, and skeletal abnormalities. Among the 18 increased NT pregnant women, 12 patients (67%) had Trisomy 21. Compared to normal fetuses of the same gestational age, the majority of fetuses with DS have an increased NT measurement [31].Furthermore, increased NT is attributed to aortic isthmus narrowing, cardiovascular defects which cause overperfusion of the head and neck, or abnormal/delayed development of the lymphatic system [32]. Some studies have found 36% of DS had congenital malformations. Cardiac defects were the most common malformation [33]. But considering the sample size is small, the relationship cannot be confirmed confidently, thus the studies need further research include a greater number of patients.

## Conclusion

We found that the rate of fetal chromosomal abnormalities was 4.12% through amniocentesis. The serological screening and NIPT that were included in the prenatal screening methods all had false positive and false negative rates. Although they are both prenatal screening techniques, maternal serum screening cannot be replaced by NIPT. The pregnancy women should accept NIPT in a qualified prenatal diagnostic center. We recommend that pregnant women at high or critical risk undergoing prenatal screening should confirm the fetal karyotype through amniocentesis. Moreover, if women receive a positive result via NIPT, they should not have a pregnancy termination without undergoing further prenatal diagnosis.

## Materials and methods

### Sample collection

We evaluated 4953 16- to 23-week singleton gestation cases for amniocentesis, who attended the outpatient abortion clinic of the Prenatal Diagnosis departments of the First Hospital of Changchun, Jilin Province, Northeastern China, between November 14, 2012 and December 12, 2018. This study was approved by the ethics committee of the hospital (No.2012–115), and all patients provided informed consent to participate in the study.

The amniocenteses were for indications including advanced AMA, a positive NIPT result, a positive aneuploidy screening result, abnormal ultrasound, history of abnormal pregnancy, and paternal/maternal carriers. We enrolled 4952 samples out of 4953 amniocentesis samples except for one karyotyping showed trisomy 18, however the FISH result was normal. Because some pregnant women involved two or more indications, the 4952 participants contained 5254 indications. Clinical information was mostly obtained from their clinicians using a questionnaire. A total of 3583 participants received serological screening, 97 had NIPT, 309 had ultrasonography and 1265 participants were classed as AMA. To analyze the possible relationship between maternal age and abnormal embryo development, we further divided the women providing samples into five age groups (< 25, 25–29, 30–34, 35–39, and ≥ 40 years).

### Karyotype analysis of amniotic fluid cells

Amniotic fluid cells were obtained by amniocentesis at 16–23 weeks of gestation. They were cultured in CHANG Amnio® Medium (Irvine Scientific, Santa Ana, CA, USA), followed by treatment with colcemid. A total of 4953 amniocentesis samples were processed. G-banding of metaphase chromosomes was performed by standard methods [34]. For each individual, a minimum of 30 metaphase cells was counted and at least five cells were analyzed. Chromosome abnormalities were described according to the criteria established by the International System for Human Cytogenetic Nomenclature [34].

### FISH analysis

FISH was performed using commercially available whole-chromosome painting probes for chromosomes 13, 18, 21, X and Y. FISH was carried out using centromeric probes for chromosomes 18, X and Y (CSP18-Spectrum blue, CSPX-Spectrum green and CSPY-Spectrum red, Beijing GP Medical Technologies, Beijing, P. R. China). The chromosome 13- and 21- specific probes (GLP13- Spectrum green and GLP21-Spectrum red, respectively; Beijing GP Medical Technologies) were used. Chromosome denaturation, hybridization, and signal detection were done as described by Luo et al. [35].

### NIPT analysis

With the continually evolving of next-generation sequencing technologies, NIPT also has been applied in several sequencing platforms such as a semiconductor sequencing platform [36, 37], Illumina sequencing platform [37, 38], and the Beijing Genomics Institute (BGI) sequencing platform [39].

Maternal peripheral blood was collected in blood collection tubes and processed within the required time. Student’s t test was performed based on null/alternative hypotheses, and the relative logarithmic likelihood odds ratio was subsequently calculated. Absolute z-score > 3 and L score > 1 were used as warning criteria [40–42].

### Prenatal serological screening analysis

The first-trimester combined screening (FTS) was performed at 11–13^+ 6^ weeks of gestation using maternal age, fetal NT thickness and concentrations of maternal serum free β-hCG and pregnancy-associated plasma protein-A (PAPP-A) for risk calculation. The second-trimester triple screening (STS) was performed at 15–20^+ 6^ weeks of gestation using maternal age and maternal serum concentrations of AFP, free β-hCG and unconjugated estriol (uE3) for risk calculation. Gestational age was determined by fetal crown rump length (CRL) or biparietal diameter (BPD). In all cases, maternal serum was sampled at 11–13^+ 6^ or 15–20^+ 6^ weeks of gestation after obtaining informed written consent. The ultrasound examination and the maternal blood extraction for FTS were performed on the same day. The serum marker levels of FTS and STS were measured by AutoDELFIA (PerkinElmer, Finland). Both marker levels and NT thickness were converted into multiple of the median (MoM), which is used to calculate the risk of chromosomal abnormality, base on gestational age. As well as information on earlier pregnancy with DS, maternal weight, maternal age and smoking habits were taken into account for risk calculation on DS. DS screen-positive at a term risk cut-off of 1 in 270 for both first and second trimester tests, and ES risk rate < 1/350.

## Data Availability

The data used to support the findings of this study are included within the article.

## Abbreviations

ACMG: American College of Medical Genetics and Genomics
AFP: Alpha fetoprotein
AMA: Advanced maternal age
BPD: Biparietal diameter
CRL: Crown rump length
DS: Down’s syndrome
ES: Edwards syndrome
FISH: Fluorescence in situ hybridization
FTS: First-trimester combined screening
HCG: Human chorionic gonadotropin
NF: Neck fold
NIPS: Non-invasive Prenatal Screening
NIPT: Non-invasive prenatal testinghas
NT: Nuchal translucency
PAPP-A: Pregnancy-associated plasma protein-A
STS: Second-trimester triple screening
uE3: Unconjugated estriol
